# The impact of frailty on glycaemic control in older adults with type 1 diabetes: An observational study

**DOI:** 10.1111/dme.70406

**Published:** 2026-06-23

**Authors:** Jonathan A. Golding, Scarlett O. Long, Suzy V. Hope, Juliet E. Wright, Ali J. Chakera, Tom J. Levett

**Affiliations:** ^1^ University Hospitals Sussex NHS Foundation Trust Brighton UK; ^2^ Brighton and Sussex Medical School Brighton UK; ^3^ Royal Devon University Healthcare NHS Trust Exeter UK; ^4^ University of Exeter Exeter UK; ^5^ University of Surrey Guildford UK

**Keywords:** frailty, hypoglycaemia, older adults, type 1 diabetes

## Abstract

**Aims:**

The type 1 diabetes population is ageing. Frailty is the age‐related physiological decline that occurs across organ systems, increasing the body's vulnerability to stressors. Current consensus‐based guidelines, derived from evidence in type 2 diabetes, prioritise hypoglycaemia avoidance, which increases falls, hospitalisation and mortality risk in older adults. This study assesses whether frailty is associated with increased hypoglycaemia in older adults with type 1 diabetes.

**Materials and Methods:**

Participants were over 65, with type 1 diabetes, confirmed by C‐peptide measurement. Frailty was categorized as robust, prefrail or frail as per Fried Frailty Phenotype (gait speed, weight loss, hand‐grip strength, exhaustion and physical activity). Secondary analyses used Clinical Frailty Scale and Short Physical Performance Battery. Cognition was assessed using Montreal Cognitive Assessment score (MoCA). Blinded continuous glucose monitors (CGM), Dexcom G6/G7, were worn for 10 days. CGM metrics were compared between frailty categories, focusing on percentage time below range (%TBR).

**Results:**

83 participants met the inclusion criteria. 38 were robust (45.8%), 29 prefrail (34.9%) and 16 frail (19.3%). Frail participants were older, with more comorbidities and diabetes complications. Frailty was associated with higher HbA1c (*p* = 0.025) and lower MoCA scores (*p* = < 0.001). There was no difference between any CGM‐derived glycaemic parameter across the frailty spectrum, regardless of frailty assessment used.

**Conclusions:**

While frailty is often associated with hypoglycaemia in diabetes, this was not demonstrated in our type 1 diabetes cohort. Raised HbA1c levels with frailty may reflect recommendations to relax glucose targets. However, no difference was found in %TBR with frailty.

**What's new?**

**What is already known?**

The type 1 diabetes population is ageing, and frailty can present a clinical challenge. Previous research into diabetes and ageing has focussed on type 2 diabetes.

**What has this study found?**

We found no difference in the occurrence of hypoglycaemia with frailty status.

**What are the implications of this study?**

Older adults with type 1 diabetes can self manage successfully despite the challenges of frailty. Further research should look at other challenges that come with ageing, including cognitive impairment.

## 1 INTRODUCTION

Between 1990 and 2019 the global prevalence of type 1 diabetes in people aged over 65 increased by 180%.^1^ There are now over 1.1 million people living with type 1 diabetes over 60 years old.^2^ This demographic shift has been facilitated by advances in diabetes care over recent decades which have allowed people living with type 1 diabetes to live longer. In high‐income countries continuous glucose monitors (CGM) and insulin pumps are now mainstays of treatment. As advancements facilitate tighter glycaemic control earlier in life, the older adult type 1 diabetes population is projected to increase further.^2^

The challenges of living with type 1 diabetes through older age can include the onset of geriatric syndromes, such as frailty, which may complicate glycaemic management through alterations to appetite, weight activity levels and mood. Frailty is commonly defined as: ‘A physiological decline and loss of reserve across organ systems that leaves an individual vulnerable to stressors which trigger deteriorations in health or functional status’.^3^ Diabetes has been shown to be a risk factor for frailty development.^4^ Unfortunately, little research addresses frailty in older adults with type 1 diabetes, meaning clinical guidelines cannot make evidence‐based recommendations.^5^

More evidence exists in older adults with type 2 diabetes, where hypoglycaemia has been linked to increased harm from falls, fractures, hospitalisations and overall mortality.^6^ Additionally, there is reduced time to derive benefit from tight glucose control in older adults, especially with co‐existing frailty. Consensus guidelines therefore recommend relaxing glycaemic targets as frailty progresses to prioritise hypoglycaemia avoidance.^7^, ^8^

In the absence of evidence, guidelines designed for type 2 diabetes are often extrapolated to be used for older adults living with type 1 diabetes. It is commonly assumed that frailty impacts type 1 diabetes in the same way, despite clear differences in aetiology. This is an important clinical consideration that requires investigation.

## 2 MATERIALS AND METHODS

The primary objective was to assess whether frailty is associated with more hypoglycaemia in older adults with type 1 diabetes. Secondary objectives were to see whether frailty, age or cognition are associated with changes to other glycaemic parameters. A pragmatic sample size of 100 participants was proposed, as insufficient data made sample size calculations impossible.

### 2.1 Overview of methods

This prospective cross‐sectional observational study, sponsored by University Hospitals Sussex, was conducted at six participating sites in the United Kingdom (UK). Ethical approvals were obtained from the Health Regulation Authority and Research Ethics Committee. The study was registered on the clinical trial registry, ISRCTN30513341 and took place between September 2022 and July 2024. Each participant gave informed written consent.

### 2.2 Participants

Participants were eligible if they had an established clinical diagnosis of type 1 diabetes for at least 3 years. Random C‐peptide (urine or blood) was used to confirm type 1 diabetes in all participants. Participants were excluded if their serum C‐peptide was > 200 pmol/L or their urine C‐peptide:creatinine ratio was > 0.2 nmol/mmol. Participants were excluded if they lacked capacity to understand or comply with the trial procedures. Eligibility criteria were intentionally broad to encourage inclusion.

### 2.3 Study procedures and data collection

The study took place in a clinic, or at the participant's residence, according to participant preference. Clinical and demographic data were collected including age; gender; ethnicity; medical history; diabetes history (including diabetes duration and complications); social and living circumstances. The following validated questionnaires were completed: Gold and Clarke scores to assess hypoglycaemia awareness^9^, ^10^; Diabetes Distress Questionaire‐17 (DDS‐17)^11^; Montreal Cognitive assessment (MOCA)^12^; EQ‐5D‐5L to assess quality of life; and the Lawton Instrumental Activities of Daily Living scale to assess for functional dependence.^13^, ^14^

Three different frailty assessments were used. There is no uniformly agreed frailty assessment in the research literature. The use of three commonly used frailty assessments improves the comparability with other research:

### 2.4 The fried frailty phenotype (FFP)

FFP assesses five domains (hand grip strength, gait speed, unintentional weight loss, exhaustion, physical activity).^3^ Each domain scores 1 if present or 0 if absent, with a total score of 5 possible. Individuals were considered robust with a score of 0, prefrail with a score of 1 or 2 and frail with a score of 3 or more.

### 2.5 Clinical frailty scale (CFS)

CFS is based upon the subjective assessment of the clinician, taking into account two questionnaires: the EQ‐5D‐5L and the Lawton scale.^15^ Data were grouped into not frail (CFS 1–4) and frail (CFS ≥ 5).

### 2.6 The short physical performance battery (SPPB)

The SPPB is purely a measure of physical performance across three physical tests (gait speed, balance test, sit‐to‐stand test).^16^ Each test carries a possible score of four, giving a total possible score of 12 with higher scores representing better performance. A score of 10–12 is considered good performance, 7–9 moderate performance and ≤ 6 poor performance.

### 2.7 Cognitive assessment

A sub analysis looked at CGM metrics in relation to cognitive function. The MOCA was used for cognitive assessment.^12^ Scores of ≥ 26 were considered normal; 20–25 was considered mild cognitive impairment; and scores of ≤19 were considered severe cognitive impairment. For some analyses participants were grouped as normal (≥ 26) or impaired (< 26). The full MoCA score requires sight. An adapted version is available for individuals with sight‐loss without the visuospatial executive or naming sections, with a total possible score of 22. Previous studies have provided norms and score‐equivalence across the two tests.^17^ Based on this, blind MoCA scores of ≥ 19 were considered normal, 14–19 was considered mild impairment and ≤ 13 were considered severe impairment.

All participants were fitted with a CGM, in blinded mode (Dexcom G6/G7) and used in accordance with the manufacturer's instructions, worn for 10 days. Participants were given a receiver and instructed to keep it within six metres whenever possible. All alarms were turned off. Individuals already using open CGM wore the research device in addition. Anonymised data from the CGM were uploaded onto a secure online database (Dexcom Clarity) and linked with their clinical and demographic data, which was on a separate secure database (REDCap).^18^, ^19^ All participants underwent blood testing for HbA1c and full blood count.

### 2.8 CGM analysis

Completed data were extracted from Clarity and imputed into EASY GV (Version 10), a validated software for CGM analysis.^20^ The percentage time below range (%TBR) of individuals across different frailty levels was compared. %TBR refers to the percentage of time the glucose levels are below 70 mg/dL (3.9 mmol/L). A second %TBR is reported which is the percentage of time the glucose levels are below 54 mg/dL (3.0 mmol/L). Secondary analysis compared percentage time‐in‐range (%TIR); percentage time‐above‐range (%TAR); percentage co‐efficient of variation (%CV); and glucose management indicator (GMI), a CGM‐metric which estimates a person's HbA1c level, across different frailty levels.^21^ A summary table of the definitions of CGM metrics used can be found in Table S1.

### 2.9 Data management

Data were collected on anonymised paper case report forms (CRFs) and transcribed into the online database at the earliest opportunity before paper CRFs were destroyed. Statistical analysis used IBM SPSS Statistics (version 29).

### 2.10 Presentation of the data and statistical analysis

Normally distributed data are presented as mean ± standard deviation (SD). Skewed data are presented as median [lower quartile, upper quartile]. Categorical data are presented as *n* (%). Differences between groups were assessed using *t*‐test, ANOVA, Chi square or Kruskal–Wallis tests depending on the distribution and grouping of data. Where the frequencies were low, the Fisher's exact test was used. Statistical significance was considered with a *p* value < 0.05.

## 3 RESULTS

86 participants were recruited. Three participants had a C‐peptide above the pre‐determined cut‐off and were excluded.

### 3.1 Description of the data

Mean age was 75.5 years ±6.06, with median diabetes duration of 46 years [30, 58]. 43 (51.8%) participants were men with the remainder women. 82 participants were White European, one participant of Asian ethnicity. 86.7% were using open CGM. 10 were using insulin pumps, including three using Hybrid Closed Loop (HCL).

CGM data capture was good with a median CGM active time of 90%. When assessed by FFP, 38 (45.8%) were assessed as robust, 29 (34.9%) prefrail and 16 (19.3%) frail.

Details on differences between the participants based on frailty status are shown in Table 1. Frailty was associated with advancing age, falls, increased comorbidity, polypharmacy and lower cognitive performance. Frailty was associated with longer diabetes duration and diabetes‐related complications.

**TABLE 1:** Descriptive summary by frailty category—Fried frailty phenotype.

Characteristic	Full population *n* = 83	Robust *n* = 38	Prefrail *n* = 29	Frail *n* = 16	*p*
Age (years)	75.5 ± 6.06	74.2 ± 4.54	75.0 ± 7.13	79.8 ± 5.47	**0.050**
Diabetes duration (years)	46 [30, 58]	43.5 [34, 56]	42 [30, 57]	59 [34.5, 65]	**0.015**
BMI (kg/m^2^)	26.0 ± 4.2	25.3 ± 3.5	27.0 ± 4.2	25.7 ± 5.5	0.258
Male	43 (51.8%)	24 (63.2%)	12 (41.4%)	7 (43.8%)	0.162
12 or more years of formal education	31 (37.3%)	13 (34.2%)	12 (41.4%)	6 (37.5%)	0.774
**Ethnicity/race**					
White, European	82 (98.8%)	37 (97.4%)	29 (100%)	16 (100%)	0.530
**Medical history**					
Dementia diagnosis	3 (3.6%)	1 (2.6%)	0 (0%)	2 (12.5%)	N/A
No of comorbidities	4 [2, 6]	2 [2, 4]	5 [3, 5.5]	6.5 [5.25, 8.75]	**< 0.001**
One or more falls in the last year	30 (36.1%)	7 (18.4%)	14 (48.3%)	9 (56.3%)	**0.005**
Polypharmacy (≥ 5 medications in addition to insulin)	45 (51.7%)	14 (36.8%)	17 (58.6%)	14 (87.5%)	**0.002**
**Diabetes complications (self reported)**					
Ischaemic Heart Disease	12 (14.5%)	3 (7.9%)	3 (10.3%)	6 (37.5%)	**0.026**
Stroke/TIA	10 (12.0%)	1 (2.6%)	5 (17.2%)	4 (25.0%)	N/A
Nephropathy	14 (16.9%)	2 (5.3)	4 (13.8%)	8 (50.0%)	**< 0.001**
Retinopathy	11 (13.3%)	2 (5.3%)	3 (10.3%)	5 (31.3%)	N/A
History of foot ulceration	15 (18.1%)	1 (2.6%)	7 (24.1%)	7 (70.0%)	**< 0.001**
Amputation	2 (2.4%)	0 (0%)	0 (0%)	2 (12.5%)	N/A
**Diabetes demographics**					
CGM use	72 (86.7%)	34 (89.5%)	25 (86.2%)	13 (81.3%)	0.714
Insulin pump	10 (12.0%)	4 (10.5%)	5 (17.2%)	1 (6.3%)	0.515
Hybrid closed loop	3 (3.6%)	2 (5.3%)	1 (3.4%)	0 (0%)	N/A
Help to check glucose	6 (7.2%)	1 (2.6%)	0 (0%)	5 (31.3%)	N/A
Help to give insulin	4 (4.8%)	0 (0%)	0 (0%)	4 (25.0%)	N/A
HbA1c, mmol/mol (%)	61.4 ± 12.6 (7.8 ± 3.3)	58.6 ± 10.9 (7.5 ± 3.1)	61.1 ± 12.1 (7.7 ± 3.3)	68.7 ± 15.1 (8.4 ± 3.5)	**0.025**
**Reduced hypoglycaemia awareness**					
Clarke score ≥ 4	15 (18.1%)	3 (7.9%)	6 (20.7%)	6 (37.5%)	**0.034**
Gold score ≥ 4	24 (28.9%)	9 (23.7%)	10 (34.5%)	5 (31.3%)	0.622
**Diabetes distress**					
DDS‐17 score of ≥ 3	5 (6.0%)	0 (0%)	3 (10.3%)	2 (12.5%)	0.058
**Living circumstances**					
Lives alone	27 (32.5%)	10 (26.3%)	7 (24.1%)	10 (62.5%)	**0.017**
Requires package of care (not residential care)	4 (4.8%)	0 (0%)	1 (3.4%)	1 (6.3%)	N/A
Care/Nursing home resident	2 (2.4%)	0 (0%)	0 (0%)	2 (12.5%)	N/A
Uses walking aids	29 (34.9%)	3 (7.9%)	11 (38.0%)	15 (93.8%)	**< 0.001**
^a^ **Cognition**					
MoCA score	24.8 ± 3.9	26.3 ± 2.1	25.7 ± 2.4	21.6 ± 4.3	**< 0.001**

*Note*: Descriptive summary by frailty category—Fried frailty phenotype. Bold values are the ones where statistical significance was met.

^a^ One participant declined cognitive assessment.

### 3.2 CGM analysis by frailty status

Each of the frailty assessments produced similar results (Table 2). For each, there was a statistically significant association between increasing frailty and higher HbA1c. However, this was not reflected by CGM metrics, with no statistically significant differences across any parameter. There was no difference in the primary outcome, %TBR < 3.9 with frailty using any frailty assessment. This result for FFP is graphically represented in Figure 1.

**TABLE 2:** Glycaemic metrics compared by frailty category.

	Robust *n* = 37	Prefrail *n* = 29	Frail *n* = 16	*p*
**Frailty assessed by FFP** ^a^				
HbA1c mmol/mol (%)	61.4 ± 12.6 (7.8 ± 1.2)	59.3 ± 11.7 (7.6 ± 1.1)	67.9 ± 13.4 (8.4 ± 1.2)	**0.025**
%TBR < 70 mg/dL	1.45 [0.40, 4.56]	1.47 [0.31, 4.22]	1.29 [0.44, 7.33]	0.766
%TBR < 54 mg/dL	0.24 [0.00, 0.95]	0.24 [0.00, 0.91]	0.28 [0.00, 1.09]	0.825
CGM active time (%)	90 [87, 92.5]	90 [87, 94]	91 [86, 92]	0.218
GMI mmol/mol (%)	58.4 ± 10.4 (7.5 ± 1.0)	57.5 ± 9.8 (7.4 ± 0.9)	60.8 ± 11.8 (7.7 ± 1.1)	0.127
Mean glucose, mg/dL (mmol/L)	175 ± 38 (9.7 ± 2.1)	171 ± 36 (9.5 ± 2.0)	180 ± 43 10.0 ± 2.4	0.241
Standard deviation	3.33 ± 0.91	3.3 ± 0.9	3.5 ± 1.0	0.160
%CV	34.6 ± 7.1	34.4 ± 6.7	35.3 ± 8.3	0.330
%TAR > 180 mg/dL	39.5 ± 20.4	38.2 ± 20.7	43.4 ± 19.5	0.270
%TIR	56.3 ± 19.2	57.8 ± 19.5	51.7 ± 17.9	0.176

	Not frail (CFS 1–4) *n* = 61	Frail (CFS ≥ 5) *n* = 21	*p*
**Frailty assessed by CFS**			
HbA1c mmol/mol (%)	59.3 ± 11.7 (7.6 ± 1.1)	67.9 ± 13.4 (8.4 ± 1.2)	**0.008**
%TBR < 70 mg/dL	1.47 [0.31,4.22]	1.29 [0.44,7.33]	0.690
%TBR < 54 mg/dL	0.24 [0.00, 0.91]	0.28 [0.00, 1.09]	0.813
CGM active time (%)	90 [87, 94]	91 [86, 92]	0.688
GMI mmol/mol (%)	57.5 ± 9.8 (7.4 ± 0.9)	60.8 ± 11.8 (7.7 ± 1.1)	0.223
Mean glucose, mg/dL (mmol/L)	171 ± 36 (9.5 ± 2.0)	180 ± 43 (10.0 ± 2.4)	0.371
Standard deviation	3.3 ± 0.9	3.5 ± 1.0	0.357
%CV	34.4 ± 6.7	35.3 ± 8.3	0.580
%TAR > 180 mg/dL	38.2 ± 20.7	43.4 ± 19.5	0.322
%TIR	57.8 ± 19.5	51.7 ± 17.9	0.202

Characteristic	Good performance *n* = 37	Moderate performance *n* = 24	Poor performance *n* = 21	*p*
**Frailty assessed by SPPB**				
HbA1c mmol/mol (%)	56.6 ± 10.4 (7.3 ± 1.0)	62.8 ± 9.5 (7.9 ± 0.9)	68.6 ± 16.0 (8.4 ± 1.5)	**0.002**
%TBR < 70 mg/dL	1.49 [0.72, 5.14]	1.32 [0.10, 3.83]	1.29 [0.37, 7.33]	0.637
%TBR < 54 mg/dL	0.25 [0.05, 0.90]	0.18 [0.00, 0.90]	0.24 [0.00, 1.09]	0.772
CGM active time (%)	90 [87, 95]	91 [87, 94]	90 [86, 91]	0.933
GMI mmol/mol (%)	56.1 ± 8.4 (7.3 ± 0.8)	58.6 ± 9.1 (7.6 ± 0.8)	62.1 ± 13.6 (7.8 ± 1.2)	0.111
Mean glucose, mg/dL (mmol/L)	166 ± 31 (9.2 ± 1.7)	175 ± 32 9.7 ± 1.8	189 ± 52 10.5 ± 2.9	0.070
Standard deviation	3.2 ± 0.9	3.2 ± 0.8	3.7 ± 0.9	0.121
%CV	34.7 ± 7.3	33.4 ± 5.6	35.9 ± 8.3	0.505
%TAR > 180 mg/dL	35.2 ± 19.2	39.8 ± 19.6	46.8 ± 22.9	0.113
%TIR	60.5 ± 18.5	56.8 ± 18.3	48.2 ± 19.8	0.062

*Note*: Glycaemic metrics compared by frailty category. Bold values are the ones where statistical significance was met.

^a^ CGM data contain 82 datasets as 1 participant's CGM data were lost.

**FIGURE 1:**
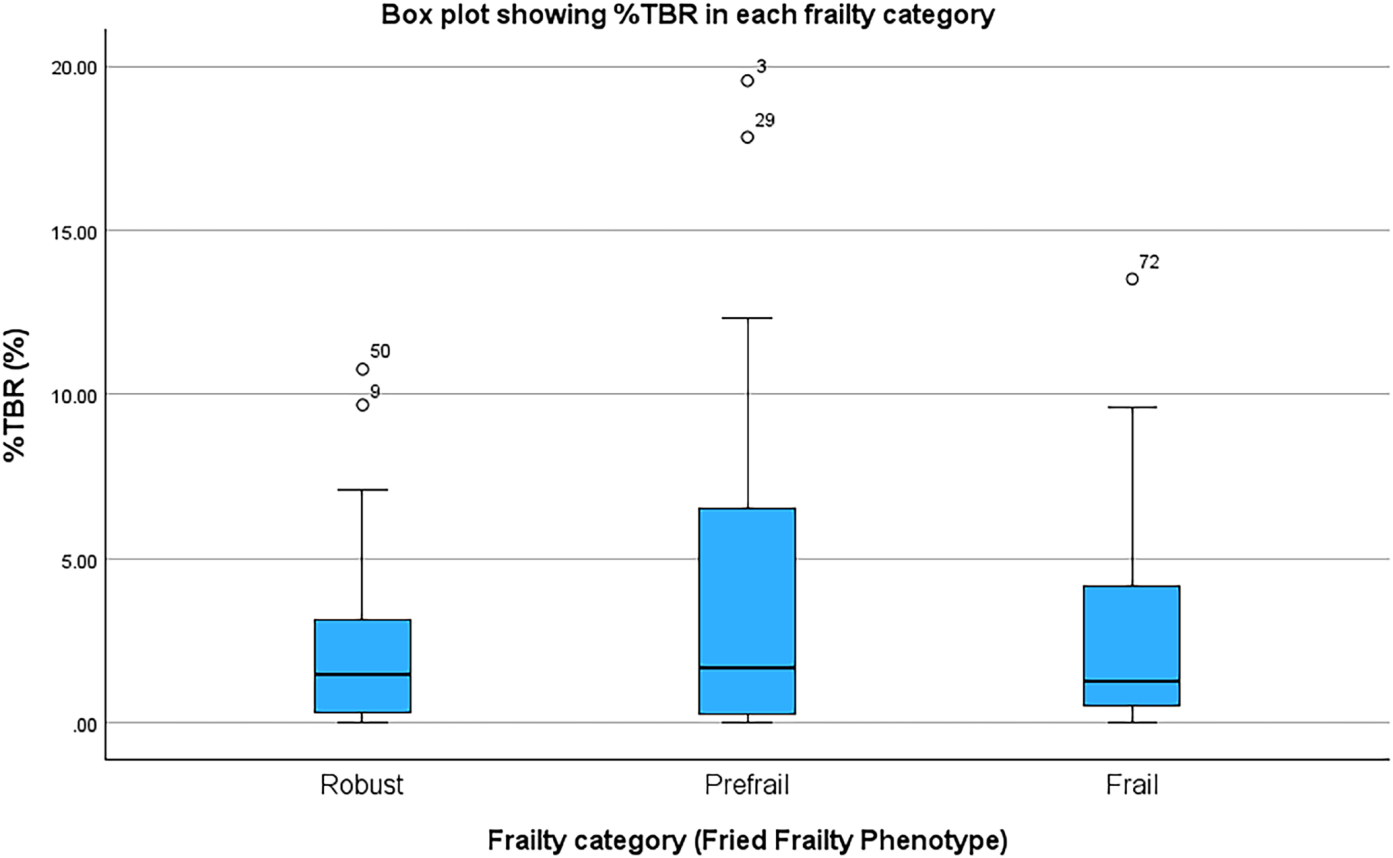
Box plot showing %TBR in each frailty category.

### 3.3 Analysis by cognitive status

It was considered possible that cognition could have a greater impact on glycaemic control than frailty. Table 3 shows the data according to MoCA score.

**TABLE 3:** Glycaemic metrics compared by cognitive scores (MoCA).

Category	Normal cognition *n* = 49	Impaired cognition *n* = 32	*p*
HbA1c mmol/mol (%)	58.6 ± 10.8 (7.5 ± 1.0)	65.9 ± 14.2 (8.2 ± 1.3)	**0.010**
%TBR < 70 mg/dL	1.4 [0.5, 4.6]	1.5 [0.4, 5.2]	0.838
%TBR < 54 mg/dL	0.2 [0.0, 1.0]	0.2 [0.0, 0.9]	0.953
CGM active time (%)	90 [85, 93]	91 [87, 92]	0.411
GMI mmol/mol (%)	55.8 ± 8.1 (7.3 ± 0.7)	62.3 ± 12.4 (7.9 ± 1.1)	**0.006**
Mean glucose, mg/dL (mmol/L)	166 ± 31 (9.2 ± 1.7)	187 ± 47 (10.4 ± 2.6)	**0.013**
Standard deviation	3.2 ± 0.9	3.5 ± 0.9	0.104
%CV	34.7 ± 7.1	34.6 ± 7.2	0.971
%TAR > 180 mg/dL	34.9 ± 18.4	46.7 ± 22.0	**0.011**
%TIR	60.7 ± 17.3	49.4 ± 20.7	**0.009**

*Note*: Glycaemic metrics compared by cognitive scores (MOCA). Bold values are the ones where statistical significance was met.

HbA1c was higher in individuals who had impaired cognition. Higher glucose levels were also demonstrated in CGM metrics by statistically significant increases in GMI, mean glucose, and %TAR and reduction in %TIR. No difference was seen in %TBR < 3.9. There was no change in glycaemic variability with cognition.

### 3.4 Analysis by age

A comparison was also made by age category (Table 4). A statistically significant increase in HbA1c was seen with older age categories (*p* = 0.008). There was no change seen in any CGM metrics.

**TABLE 4:** Analysis of CGM metrics by age category.

Characteristic	Age 65–69 *n* = 15	Age 70–74 *n* = 20	Age 75–79 *n* = 30	Age ≥ 80 *n* = 18	*p*
HbA1c mmol/mol (%)	55.7 ± 10.2 (7.2 ± 0.9)	56.9 ± 9.6 (7.4 ± 0.9)	63.5 ± 11.7 (8.0 ± 1.1)	68.1 ± 12.6 (8.4 ± 1.2)	**0.008**
%TBR < 70 mg/dL	3.3 [0.7, 6.4]	2.1 [0.2, 5.9]	1.3 [0.5, 2.2]	1.2 [0.2, 6.3]	0.510
%TBR < 54 mg/dL	0.4 [0.0, 1.4]	0.3 [0.0, 1.1]	0.2 [0.0, 0.7]	0.3 [0.0, 2.1]	0.858
CGM active time (%)	90 [81, 94]	90 [89, 92]	91 [87, 94]	91 [82, 96]	0.853
GMI Mmol/mol (%)	53.8 ± 8.3 (7.1 ± 0.8)	56.0 ± 9.8 (7.3 ± 0.9)	60.2 ± 10.2 (7.7 ± 0.9)	62.5 ± 11.5 (7.9 ± 1.1)	0.059
Mean glucose, mg/dL (mmol/L)	158 ± 29 8.8 ± 1.6	166 ± 38 9.2 ± 2.1	180 ± 38 10.0 ± 2.1	187 ± 45 10.4 ± 2.5	0.116
Standard deviation	3.0 ± 0.8	3.2 ± 1.0	3.5 ± 0.8	3.3 0.9	0.375
%CV	33 ± 6	35 ± 7	35 ± 6	34 ± 9	0.916
%TAR > 180 mg/dL	30.7 ± 18.9	35.2 ± 22.5	42.8 ± 17.4	46.7 ± 21.6	0.083
%TIR	64.9 ± 17.9	59.9 ± 21.1	53.8 ± 16.2	49.0 ± 20.7	0.081

*Note*: Bold values are the ones where statistical significance was met.

## 4 DISCUSSION

### 4.1 The impact of frailty on glycaemia

No statistically significant differences in any CGM metrics were found between frailty levels. There was no difference in %TBR across the frailty spectrum regardless of the frailty assessment used.

HbA1c was found to increase with frailty alongside a non‐significant increase in glucose levels in CGM metrics with increasing frailty, suggesting frailty is associated with higher glucose levels. Cross‐sectional design makes it impossible to assess causation, although it may reflect clinical guidelines for older adults, which promote relaxed glycaemic targets.^7^, ^8^

Consideration was given to whether frailty may be associated with increased glucose variability, which could account for the reason %TBR did not reduce, despite the suggestion that glucose levels were higher in the frail group. However, no difference was seen in SD or %CV with frailty.

No previous studies have assessed CGM metrics alongside frailty in older adults with type 1 diabetes. However, similar studies have been conducted in individuals with type 2 diabetes.^22^, ^23^ One notably only included individuals on insulin.^22^ Results showed that frailty was associated with higher glucose levels (increased mean glucose, GMI and %TAR). %TBR was lower in older adults living with frailty (1.3% [0.0, 4.6] in least frail group, 0.0% [0.0, 1.2] in most frail group, *p* = 0.017). Our study in type 1 diabetes similarly suggests increased glucose levels with frailty, but without reduction in hypoglycaemia.

One factor that could have influenced our findings is the high prevalence of open CGM use (86.7%). Older adults were expected to have a low uptake of CGM due to potential barriers to accessing technology.^24^ The role out of CGM technology was occurring rapidly during the study, and consequently, CGM uptake was better than expected. The additional glycaemic awareness that CGM provides might have led to improvements in glycaemia as insulin regimens were adapted in response. A previous trial studied the effect of open CGM on hypoglycaemia, showing significant reductions in the occurrence of hypoglycaemia in older adults living with type 1 diabetes.^25^ In that study, the baseline %TBR was 5.1% [3.0, 9.7], and after CGM use for 6 months was 2.7% [1.6, 4.6]. The %TBR in the current study was lower even than what was found in the CGM‐arm of that trial.^25^ This could reflect improvements in glycaemia that have come through experience using CGM. This is particularly relevant given that in this study, frailty was associated with impaired hypoglycaemia awareness (Clarke score).

Future studies should seek to clarify whether frailty is associated with changes in CGM metrics. Prospective studies should further assess the outcomes when therapeutic targets are pre‐defined, as studies to date have not factored this into their methodology.

### 4.2 The impact of cognition on glycaemia

When individuals with normal cognition (MoCA ≥ 26) were compared against individuals with impaired cognition (MoCA < 26), increases were seen in HbA1c, GMI, mean glucose and %TAR, while a decrease was seen in %TIR. No changes were seen in %TBR.

No previous studies have assessed CGM metrics against cognitive performance in older adults with type 1 diabetes, but a similar study was carried out in older adults with type 2 diabetes, showing similar results.^26^ These results could reflect the relaxed glucose targets recommended in cognitive impairment. Alternatively, they could reflect missed doses of insulin or hypoglycaemic agents. Further studies should clarify these findings in both type 1 and type 2 diabetes and assess the reasons behind them.

Participants in this study were excluded if their cognition was impaired so that they were unable to understand or comply with the study procedures. Consequently, there were few participants with severe cognitive impairment. The MoCA test is not diagnostic of dementia, only indicating impaired cognition. Three participants had a formal diagnosis of dementia, two of these were self managing their diabetes at home, and the third was in long‐term care but still played an active role in self management. It is likely that participants with more advanced cognitive impairments would have more extreme alterations to their glycaemic metrics, given the importance of cognition to type 1 diabetes self management.

With individuals living with type 1 diabetes for longer, it is expected that more will experience the dual diagnosis of type 1 diabetes and dementia. Further research is urgently needed to devise practical management strategies to safely manage type 1 diabetes through the onset of dementia.

### 4.3 Glycaemic targets in older adults with type 1 diabetes

It is notable that guidelines for diabetes management in older adults currently recommend target HbA1c and glucose levels for varying degrees of dependence but offer no recommendations on CGM‐derived glycaemic parameters.^7^, ^8^ Recommendations from the international consensus on time‐in‐range do suggest altered CGM targets for ‘older or high risk’ individuals with type 1 and 2 diabetes, recommending %TBR of under 1%. However, there is no differentiation based on frailty status.^27^ Longitudinal studies assessing the impact of varying levels of glycaemia in older adults with type 1 diabetes and frailty will give perspective on the risk associated with higher‐than‐normal glucose levels which are currently recommended for this group.

### 4.4 The clinical perspective

Older adults with type 1 diabetes and frailty can pose a clinical challenge. Individuals can experience variable glycaemia, associated with repeated admissions and declines in functional status. This is not necessarily evident from the results of this study. It is possible that such individuals were not recruited due to barriers to inclusion. Future studies should explore the challenges associated with diabetes self management in the context of dementia and the transfer of type 1 diabetes management to carers.^28^

It is worth considering why hypoglycaemia did not become more prevalent with increasing frailty, something that seems to occur in type 2 diabetes.^29^ In contrast to type 2 diabetes, individuals with type 1 diabetes are commonly taught from diagnosis to adjust their own insulin to accommodate different factors. Many older adults with type 1 diabetes will have adjusted their insulin in response to low glucose levels before hypoglycaemia becomes problematic. Although some individuals with type 2 diabetes adopt this approach, a larger number are not advised to make medication changes themselves, either awaiting instruction or because it is not practical or necessary. The clinicians themselves may be unaware of the presence of hypoglycaemia or the need to de‐escalate therapy as frailty progresses. Type 2 diabetes is also more commonly associated with obesity. Weight loss associated with frailty can lead to an even larger change in overall insulin requirement in the presence of high insulin resistance to start with, whereas insulin resistance is not typically a feature of type 1 diabetes. The combination of these factors may make the onset of hypoglycaemia with frailty less common in type 1 diabetes when compared to type 2 diabetes. More studies must compare the differences in how frailty impacts the management of type 1 diabetes compared with type 2 diabetes, as it is far too common for them to be grouped together within literature.

## 5 STRENGTHS AND LIMITATIONS

This study is strengthened by the prospective study design, the broad inclusion criteria and the use of blinded CGM for all participants. C‐peptide was used to confirm the presence of type 1 diabetes, meaning that data were not confounded by individuals with type 2 diabetes. The inclusion of home visits enabled participation for those with limited mobility, caring responsibilities and residents of long‐term care facilities. Involvement in research studies will always be more difficult for individuals living with frailty. This can be due to restrictive eligibility criteria or practical reasons such as mobility, cognition or increased costs. The researchers took steps to address this, but it is difficult to entirely remove this impact.

This research does have limitations. There is no uniformly accepted definition or assessment of frailty, making comparisons across research difficult. This research used three frailty assessments to help mitigate this.

Another limitation is the 10‐day CGM monitoring period. This may not be enough to truly represent an individual's glycaemic profile, with 14 days recommended as optimal.^30^ However, this was the duration of one Dexcom G6/G7 sensor and practical constraints made it difficult to monitor for longer than this. As open CGM use expands with time, it should become easier to use data from individuals' own devices. Three participants were using HCL. None of these were in the frail group. These participants would likely have a lower risk of hypoglycaemia and may have skewed the results. Overall, the sample size is small, particularly in the frail group; larger studies are needed to clarify these results.

Due to the study's location, the study population is likely to be relatively affluent. It was also not ethnically diverse. Socio‐demographic factors are known to influence glycaemia, so another study population may have found different results.^31^

## 6 CONCLUSIONS

As the population of older adults living with type 1 diabetes increases, it is vital that research addresses the challenges of ageing, including frailty.^32^ This observational study found no changes in CGM‐derived metrics across the spectrum of frailty. Importantly, there was no difference in %TBR, despite evidence of higher HbA1c. Current recommendations promote targeting higher HbA1c levels as frailty progresses to prevent the harms associated with hypoglycaemia. Frailty‐adjusted targets based on CGM metrics should be designed to help guide individuals and clinicians, supported by research that investigates the impacts of glucose levels outside the normal range in older adults with type 1 diabetes.

There is a worrying lack of research into ageing with type 1 diabetes. Addressing this gap is crucial to meet the needs of an ageing population with type 1 diabetes, large swathes of whom will encounter the challenges of ageing in the coming decades.

## AUTHOR CONTRIBUTIONS

JAG conducted the research wrote the initial manuscript. SOL assisted substantially with recruitment for the study. The remaining authors formed the supervisory team. SVH, JEW, AJC and TJL contributed to reviewing and editing the manuscript prior to submission. JAG is the guarantor of this work and, as such, had full access to all the data in the study and takes responsibility for the integrity of the data and the accuracy of the data analysis.

## FUNDING INFORMATION

Funding for this study was provided through an equipment grant from Dexcom and from Brighton and Sussex Medical School.

## CONFLICT OF INTEREST STATEMENT

None of the authors have conflicts of interest to declare.

## Supporting information


Table S1:

## Data Availability

Data are available upon request from the authors.
