# Supplementary material for: The impact of frailty on glycaemic control in older adults with type 1 diabetes: An observational study
Source: Diabet Med. 2026 Jun 23;43(10):e70406. doi: 10.1111/dme.70406 (PMC13643608; doi:10.1111/dme.70406)
Supplement: Supplementary file 1 [file DME-43-e70406-s001.docx]

**Supplementary tables**

**Supplementary table 1:**

| **CGM metric** | **Abbreviation** | **Definition** |
| --- | --- | --- |
| Percentage Time Below range <54mg/dL | %TBR <54mg/dL | Percentage of time the glucose is below 54 mg/dL (3.0mmol/L) |
| Percentage Time Below range <70mg/dL | %TBR <70mg/dL | Percentage of time the glucose is below 70 mg/dL (3.9mmol/L) |
| Percentage Time in Range | %TIR | Percentage of time when the glucose is between 70mg/dL and 180 mg/dL (3.9mmol/L and 10.0mmol/L) |
| Percentage Time Above Range >180mg/dL | %TAR >180mg/dL | Percentage of time the glucose is above 180 mg/dL (10.0mmol/L) |
| Percentage Time Above Range >250mg/dL | %TAR >250mg/dL | Percentage of time the glucose is above 250 mg/dL (14.0mmol/L) |
| Standard Deviation | SD | Measure of the variation of glucose values around the mean |
| Percentage Coefficient of Variation | %CV | A measure that expresses the standard deviation as a percentage of the mean. |
| Glucose Management Indicator | GMI | a value derived from CGM data that estimates the HbA1c level |
